# Thoracolumbar spine trauma: a guide for the FRCS examination

**DOI:** 10.1007/s00590-022-03430-9

**Published:** 2022-12-03

**Authors:** Z. Hwang, M. Abdalla, B. Ajayi, J. Bernard, T. Bishop, D. F. Lui

**Affiliations:** 1 St. George’s University of London, London, SW17 0RE UK; 2 St. George’s University Hospitals NHS Foundation Trust, London, SW17 0QT UK

**Keywords:** AO Spine, Thoracolumbar, Spine, Trauma

## Abstract

Thoracolumbar spine injuries are commonly seen in trauma settings and have a high risk of causing serious morbidity. There can be controversy when it comes to classifying thoracolumbar injuries within the spinal community, but there remains a need to classify, evaluate and manage thoracolumbar fractures. This article aims to provide a guide on classification of thoracolumbar spine injuries using the AO Spine Thoracolumbar Injury Classification System (AO TLICS).

## Introduction

Thoracolumbar spine injuries are commonly seen in trauma settings and have a high risk of causing serious morbidity [1]. Therefore, it is important that a standardised classification system exists that allows for appropriate assessment of injuries and communication between different disciplines [2]. Furthermore, it allows a knowledge discipline so that healthcare providers can easily and systematically evaluate the spinal injured patient.

A good classification system has the following characteristics:

1. Sufficient detail for an accurate diagnosis.
2. Comprehensive but simple enough to be used by non-spine specialists [3, 4].
3. Offer a management plan stratified according to grade.
4. Prognosticate based on classification.

There is much controversy when it comes to classifying thoracolumbar injuries because the highly varied presentations and patient-specific factors make developing a reliable standardised classification system challenging [5]. Hence, many studies have attempted to assess the validity and inter-observer reliability of different thoracolumbar classification systems [5].

## Timeline of classification systems

- 1938: **Watson-Jones** [6] created the first classification system based solely on the morphological characteristics of injuries (X-Ray Based).
- 1963: **Holdsworth** [7] established the association between different fracture types and neurological deficits (X-Ray and Clinical Exam).
- 1983: The precise three-dimensional visualisation of fracture morphology using computed tomography (CT based) led to a major shift in the understanding of thoracolumbar injuries. **Denis** [8] introduced the three-column concept—where stability is based upon the integrity of two of the three spinal columns—and added a hierarchical element to classifying thoracolumbar injuries: grading injuries according to their biomechanics, potential for instability and neurological involvement. This more rounded understanding of thoracolumbar injuries allowed surgeons to identify those patients that should undergo surgery and paved the way for more appropriate management of patients to reach the best outcomes.
- 1994: The surgical community criticised Denis’ lack of precision [2] and proposed the highly detailed **Arbeitsgemeinschaft für Osteosynthesefragen (AO) classification system** [9] to account for as many injury types as possible (CT or X-Ray based). Although this model provided detailed descriptions of 53 different injury patterns, it was found to be too complex and unreliable to be useful to surgeons in practice [10].
- 2005: The **Thoracolumbar Injury Classification and Severity Score (TLICS)** was proposed to address the issues with previous systems [10]. It is a simpler system based around three important aspects:
  1. Injury morphology
  2. Posterior Ligamentous Complex (PLC) integrity
  3. Patient’s neurological status

TLICS relies on X-ray, CT and magnetic resonance imaging (MRI) and provides a management algorithm (Table 1) that still requires the experienced judgement of spine surgeons to make a decision in many cases [11, 12]. TLICS fails to consider the vitally important patient-specific modifiers in tailoring the management plan to individual patients [10, 13]. Nevertheless, a literature review on the safety of TLICS concludes that it is generally safe, especially with regard to preservation or improvement of neurologic function, but questions its applicability to the treatment of stable burst fractures [14]. This was shown in the literature to give false information. Often the PLC was deemed satisfactory, but patients went on to develop kyphosis [15, 16]. It also relies heavily on MRI to determine whether the PLC or tension band is injured. MRI is not a common image modality in accident and emergency. It can be time consuming and potentially dangerous [17]. Physicians should be able to determine most information through a trauma CT and clinical examination alone.

**Table 1:** TLICS and treatment algorithm

TLICS	
*Fracture morphology*	Score
Compression Burst Rotational/translation Distraction	1 2 3 4
*PLC integrity*	
Intact Unclear Disrupted	0 2 3
*Neurological status*	
Intact Nerve root injury Complete spinal cord injury Incomplete spinal cord injury/cauda equina	0 2 2 3

Treatment recommendations	Total Score
Conservative	< 4
Surgery	> 4
Grey zone	4

## How to use the AO TLICS score

**Fig. 1:**
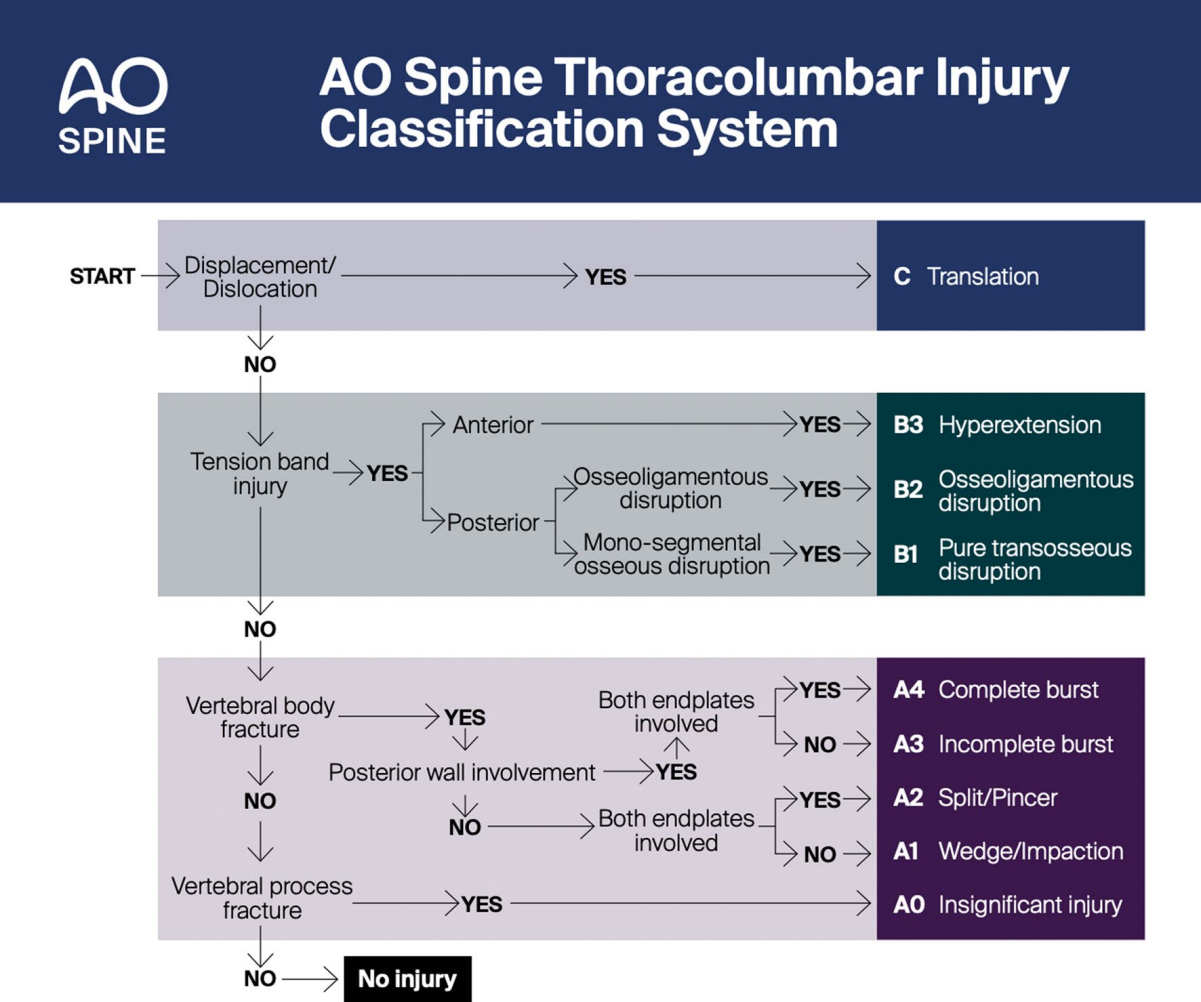
Morphological classification algorithm for AO TLICS. © AO Foundation, AO Spine, Switzerland. The AO Spine Injury Classification Systems were developed and funded by AO Spine through the AO Spine Knowledge Forum Trauma, a focused group of international spine trauma experts. AO Spine is a clinical division of the AO Foundation, which is an independent medically guided not-for-profit organisation. Study support was provided directly through the AO Spine Research Department and AO ITC, Clinical Evidence

The AO TLICS [18] is a newer classification system that strikes a balance between the overly descriptive detail of Magerl’s AO classification system [9] and addresses the weakness of the management in the TLICS score with respect to the PLC [10]. It can give an accurate diagnosis, is simple yet comprehensive and offers a level of prognostication and an algorithm [19] for managing the patient. According to the AO Algorithm (Fig. 1), one should begin by analysing the spine from the worse possible injury patterns to the least. In this way, the healthcare provider ensures that they have given due consideration to fracture patterns that they may not be familiar with and will be less likely to misdiagnose or misunderstand the degree of instability.

One should always begin by assessing the images in orthogonal views. In the past, anterior–posterior (AP) and lateral X-rays of the spine were standard. Limitations of modern pan-CT trauma scans of the whole skeleton are that doctors often forget to examine the AP or coronal plane and instead head straight for the sagittal plane and axials only.

The senior author would like to remind the reader to always assess the coronal plane to look for translation which may not be obvious in the sagittal plane. Furthermore, angulation or traumatic scoliosis needs to be appreciated with respect to biomechanical parameters of balance and remain within the Cone of Economy [20].

After Coronal images have been assessed, assess the sagittal images for translation or severe angulation. As such, Type *C* injuries represent the worst type of spinal injury and are always unstable and warrant consideration of stabilisation regardless of neurological status.

The doctor should now assess whether there is a Type *B* injury. These are distraction injuries of the tension band involving all 3 columns for those who still utilise Denis’ grading concepts. AO TLICS moves us away from the middle column philosophy, which was difficult to truly assess, to considering the spine as two columns: anterior and posterior.

*B3* injuries represent extension type injuries. They are often missed because the spine is ankylosed and the fractures with disruption of the anterior tension band may be subtle. There will be an opening wedge of the anterior column, and it may be trans-discous with very little vertebral body or posterior element injury. It is an important discipline to evaluate and consider these. These are often referred to as chalk stick fractures because the ankylosed spine resembles a long bone. Basic principles of biomechanics show that these types of injuries fare better if stabilised [18]. Conservative management is possible, but the risk of a fall leading to completion of the fracture with neurological injury is potentially significant.

Next, determine whether there is a *B*2 or *B*1 injury. A *B*2 osseoligamentous injury is a flexion type of injury which involves both anterior and posterior columns. Having a soft tissue component such as injured disc or posterior ligamentous complex is important to recognise because conservative management has a high risk of failure as soft tissue structures do not heal effectively. By default, the posterior ligamentous complex is considered disrupted.

Compare this to a *B*1 distraction injury which is essentially a pure Chance fracture [21]. Bone will heal to bone by either primary or secondary bone healing depending on the level of stability and therefore has a higher chance of uniting and restoring stability if treated conservatively. Hence the surgical algorithm for AO TLICS—the Thoracolumbar AOSpine Injury Score (TL AOSIS) (Table 2) [22]—will give this an equivocal score, allowing surgeon decision between conservative or surgical management. Factors such as polytrauma, inability to wear a Thoracolumbar Sacral Orthosis (TLSO) brace, the need for early mobilisation and pain may lead to a patient choosing surgical stabilisation over conservative treatment.

**Table 2:** AO TLICS and treatment algorithm

AO TLICS		
Morphological classification	Description	Score
*C*: translational injury	Displacement beyond physiological range of the cranial and caudal parts of the spinal column in any plane: hyperextension, translation, separation	8
*B*3: anterior tension band involvement	Disruption of the anterior longitudinal ligament (anterior tension band), extending through the vertebral body/ intervertebral disc	7
*B*2: posterior tension band involvement	Disruption of the posterior tension band with/without osseous involvement. Can affect multiple vertebrae	6
*B*1: chance	Osseous failure of posterior tension band, extending into the vertebral body. Only affects a segment of motion	5
*A*4: complete burst	Fracture involves posterior vertebral wall and both endplates	5
*A*3: incomplete burst	Fracture involves a single endplate with any involvement of posterior vertebral wall	3
*A*2: split	Fracture line involves both endplates, but not the posterior vertebral wall	2
*A*1: compression	Single endplate fracture, without posterior vertebral wall involvement	1
*A*0: spinous/transverse process	Clinically insignificant fracture of the spinous/transverse process	0
*Neurological status*		
*N*0: intact		0
*N*1: temporary	Deficit no longer present	1
*N*2: nerve root injury		2
*N*3: incomplete spinal cord injury		4
*N*4: complete spinal cord injury		4
*NX*: unreliable examination		3
*Modifiers*		
*M*1: PLC undetermined	Seems stable from bony standpoint but operative stabilisation may be considered depending on PLC integrity	1
*M*2: patient-specific health related concerns	E.g. ankylosing spondylitis, rheumatologic conditions, osteoporosis, burns, polytrauma	0

Treatment recommendations		Total Score
Conservative		< 4
Surgery		> 5
Grey zone		4, 5

AO TLICS then asks the doctor to consider the morphological fracture pattern of the vertebral body. Again, the doctor must consider the worse to best in terms of fracture pattern and stability. Both *A*4 and *A*3 are burst fractures. A burst fracture is one where the posterior wall is fractured, and there is retropulsion into the canal. *A*4 is a complete burst fracture because both endplates are fractured. *A*3 is an incomplete burst fracture and only involves one endplate. It is therefore intrinsically more stable than *A*4. It should be noted that both *A*4 and *A*3 injuries can involve the posterior elements such as pedicle, lamina, facet or spinous process. A distraction injury to the tension band may not have occurred with a severe axial loading injury and therefore not all “3-column” injuries are Type *B* injuries.

An *A*2 injury is a split in the coronal plane rather than the sagittal plane. These are important to distinguish as they can lead to non-union due to the watershed effect of the segmental vessels running in the anterior to posterior direction. A sagittal split from North to South is more likely to unite as the blood supply is unlikely to be disrupted. The coronal plane East to West split can lead to non-union and are often called cleft fractures.

*A1* injuries are simple wedge compression fractures. Whilst they can be intrinsically stable, they can be painful, and the degree of angulation can be disabling depending on where they are anatomically but can be more pronounced at the junctional level of the thoracolumbar region. One should be aware that a PLC injury can occur in combination with a “simple *A*1” injury. This would give a combined score of 4, and therefore in some patients it can be reasonable to offer surgical stabilisation and restoration or maintenance of sagittal alignment.

The neurological assessment is self-explanatory. The senior author notes the important difference between TLICS and AO TLICS. In the past, an incomplete spinal cord injury was weighted heavier than complete. They now both have the same weight of score. Incomplete and complete injuries therefore warrant equally urgent decompression and stabilisation if possible. Fehlings et al. [23] have shown that surgery within 24 h is beneficial in the improvement of ASIA grade. Lastly the doctor should be aware of *Nx*, where the patient could be obtunded. An unstable fracture pattern with an obtunded patient should give the patient the “best chance” of recovery should they have sustained a spinal cord injury, and the score of 3 reflects this to help in the decision making and management.

*M1* modifier has been briefly discussed already, and one only needs to suspect it being injured for this modifier to be applied. The reader should note the downgrade in weighting from a maximum of 3 points in the 2005 TLICS score to only 1 point now. It is utilised with the Type *A* fractures as it is already implied as part of Type *B* and *C* fractures.

*M*2 is another important modifier although it carries no weight. It does allow the physician to argue for or against surgery. In one scenario, there may be an *A*3 incomplete burst associated with a femoral fracture and flail chest with rib fractures. A polytrauma could have an *M*2 modifier grading [24]. Surgery of the femur is advised to allow stabilisation of a long bone and early mobilisation. A TLSO brace would not be recommended with a flail chest and pneumothorax. Whilst the *A*3 injury could be treated conservatively if independent, in the modern trauma setting, it would be reasonable to stabilise the fracture and restore lordosis, so a TLSO brace can be avoided, and early mobilisation can be achieved for a more efficient discharge.

Another factor, however, may show that an elderly patient has fallen down the stairs and sustained an unstable *B*2 fracture at two non-contiguous levels but also has a catastrophic head injury. Whilst it would normally be prudent to stabilise this unstable polytrauma injury, the head injury *M*2 classification would obviate surgery.

Other issues such as severe burns might also obviate the ability to proceed with surgery safely, and therefore a patient having a neurological deficit and an unstable injury cannot have safely conducted operative care due to skin coverage and infectious issues.

## Discussion

Spine surgeons are yet to universally agree on the use of a single classification system. Many studies support AO TLICS as the most complete and reliable classification system for thoracolumbar injuries [25–30]. Yet, a recent systematic review by Hwang et al. [5] concludes that the inter-observer reliability for Type *B*—particularly subtype *B*3—varies vastly and that poor reliability was demonstrated for the *A*4 subtype. AO TLICS uses CT to investigate spinal injuries, and this has good accessibility, cost-effectiveness, and sensitivity even for less experienced surgeons compared to magnetic resonance imaging (MRI) [31, 32]. However, a major concern is the high rate of PLC injury overlooked on CT alone [33]. Although it has previously been reported that MRI tends to over-diagnose PLC injury [34], a recent retrospective review of 244 patients with thoracolumbar fractures by Aly et al. [33] concludes that subsequent MRI investigation is necessary in *A*3, *A*4 and *B*2 injuries with no neurological deficit as they found that MRI significantly changed the classification and management algorithm for those injuries. Importantly, this study reiterated the requirement for an agreed definition of PLC injury on CT and MRI.

AO TLICS needs to be validated in the paediatric population, considering the unique biological aspects of bone healing and metabolism in children, and further training of classification naïve clinicians is required to improve correct diagnosis and appropriate management of injuries [5]. Although the TLICS and TL AOSIS provide safe guidelines for management of thoracolumbar injuries, indications for surgical management of injury types within the grey area must be reviewed.

## Conclusion

The authors recommend the AO TLICS score to evaluate thoracolumbar spine injuries. It is much simpler than the original AO. It is detailed enough for accurate diagnosis, management, and prognosis. It accounts for neurological status including the obtunded patient. Less weight is given to the PLC injury, and there is rightly no emphasis on utilising MRI to evaluate the traumatically injured patient. The majority of surgical decisions in trauma should be possible with the pan-CT trauma whole skeleton. Ultimately, the algorithm, as designed by the AO Group, provides an excellent knowledge discipline to carefully evaluate the spinal injured patient from the worst type of injury down to least in a systematic approach so as not to miss common pitfalls.
